# Radioiodine Therapy: Alternative for the Treatment of Complicated Thyroid Ectopia

**DOI:** 10.7759/cureus.55162

**Published:** 2024-02-28

**Authors:** Manale Otmani, Ayat Mouaden, Chaymae Bensaid, Imad Ghfir, Hasnae Guerrouj

**Affiliations:** 1 Department of Nuclear Medicine, Ibn Sina Hospital Center, Mohammed V University - Souissi, Rabat, MAR

**Keywords:** intratracheal, scintigraphy, radioiodine 131, ectopia, thyroid

## Abstract

Thyroid ectopy is the presence of thyroid tissue outside its normal cervical location. Clinical manifestations of thyroid ectopy are varied. The latter complications can be life-threatening. Emergency treatment is often surgical or endoscopic. We report a case of a 26-year-old man with tracheal thyroid ectopy, complicated by respiratory distress, in whom conventional treatments were not feasible. The patient was treated with radioiodine-131 administered in liquid form. The final control showed the complete resolution of the intra-tracheal mass. Intra-tracheal thyroid ectopy is a rare anomaly in which surgery is the traditional treatment. In certain cases where surgery is not feasible or refused, treatment with iodine-131 is a safe and effective alternative for the removal of ectopic thyroid tissue. The aim of our work is to show the significant efficiency of radioiodine therapy as an alternative for the treatment of complicated thyroid ectopia.

## Introduction

Thyroid ectopy is the presence of thyroid tissue outside its normal cervical location. Clinical manifestations of thyroid ectopy are varied and depend on whether or not the thyroid tissue is functional. Thyroid ectopy may be asymptomatic, or manifested by clinical or biological hypothyroidism. It may also cause bleeding or compressive signs in the upper aerodigestive tract [1]. The latter complications can be life-threatening. Emergency treatment is often surgical or endoscopic. We present a case of a patient with tracheal thyroid ectopy, complicated by respiratory distress, in whom conventional treatments were not feasible.

## Case presentation

A 26-year-old man with a history of undocumented total thyroidectomy at the age of six years presented to the emergency department with dyspnea and was treated as an acute asthma attack. A few hours later, the patient presented with disturbed consciousness and laryngeal stridor with sternal and intercostal stretch. Oxygen saturation was low (80%) on a high-concentration oxygen mask, with cyanosis of the extremities. The blood pressure was normal at 110/78 mmHg.

A cervicothoracic CT scan revealed a well-limited, hypodense tissue process in the thyroid cavity, enhanced after injection of contrast, measuring 34 × 37 × 22 mm, exerting a mass effect on the trachea, which was of reduced caliber. Posteriorly, it lies opposite the vertebral body of D2, respecting the separation line.

An emergency tracheotomy was performed and the patient was put in respiratory condition.

**Figure 1:**
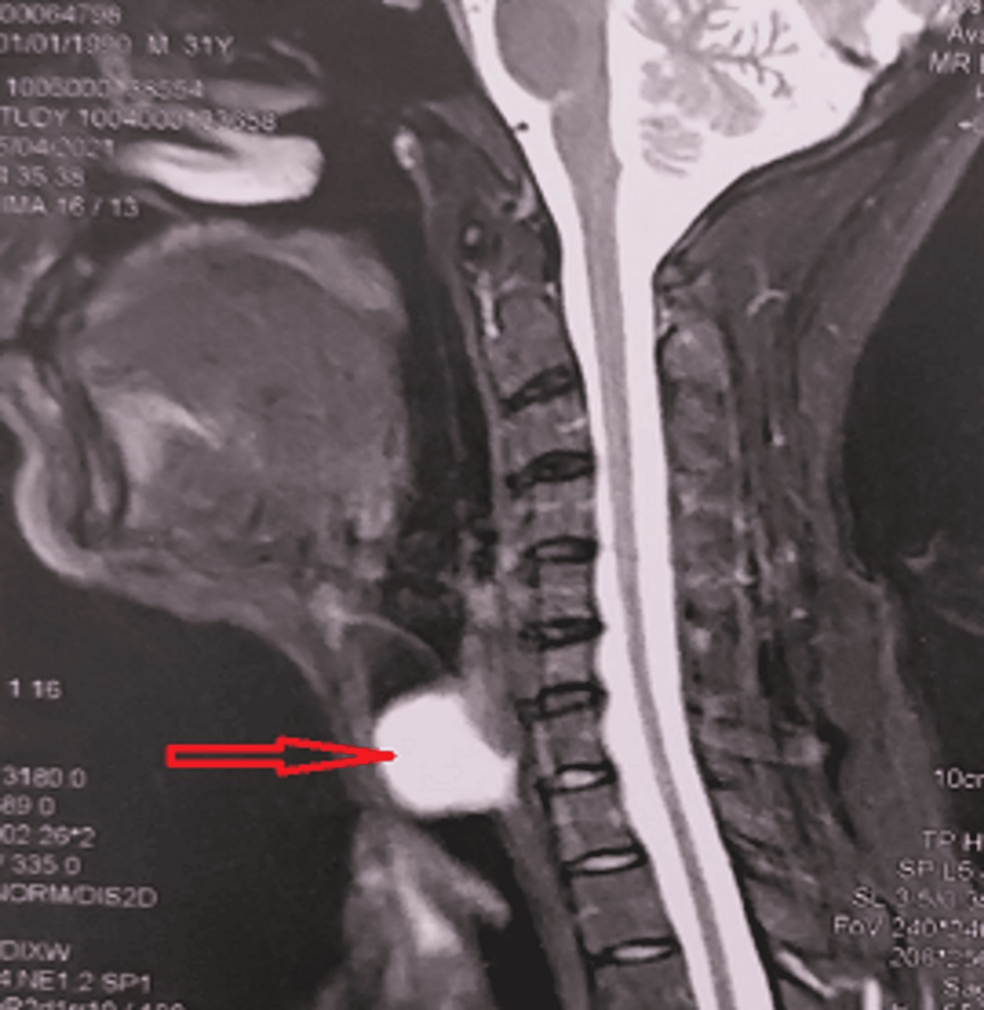
Sagittal T2 MRI showing the intra-tracheal pathologic process.

Cervical MRI revealed an endotracheal tissue lesion, with T1 hyposignal, T2 hypersignal, intensely enhanced and discreetly heterogeneous after injection of contrast, measuring 21 × 27 × 26 mm in diameter, inserted into the right lateral wall of the trachea, occupying almost the entire lumen and appearing to be continuous with the right lobe of the thyroid. The process shows the same signal characteristics of residual thyroid tissue located on either side of the cervical trachea, with the presence of another formation of the same signal characteristics located in the left retro-pharynx, measuring 11 × 19 mm, well limited, discreetly pushing back the homolateral piriform sinus (Figure 1).

A biopsy of the endotracheal process was performed, confirming the thyroid nature of the tissue.

**Figure 2:**
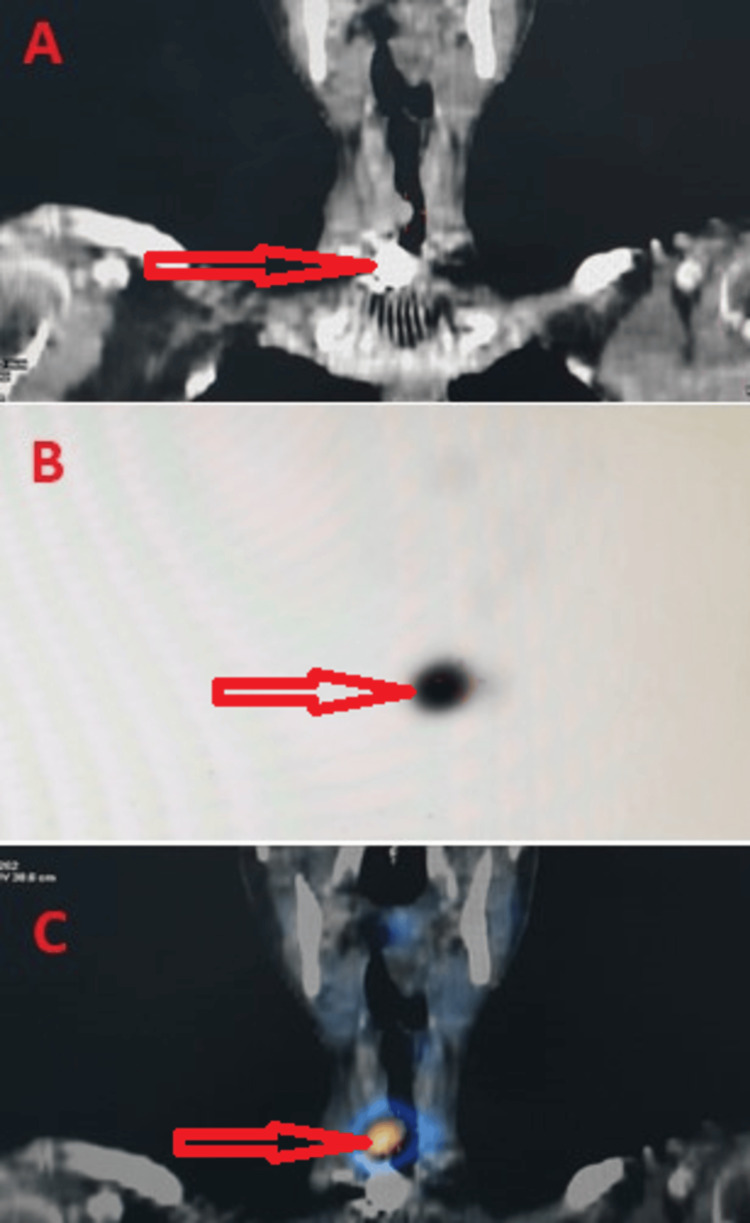
Coronal CT cervical image (A), SPECT 131I image (B), and fusion SPECT/CT image (C) showing intense uptake at the intra-tracheal pathological process. SPECT: single photon emission computed tomography.

The case was discussed in a multidisciplinary meeting between otorhinolaryngologists, thoracic surgeons, and nuclear physicians and we decided to propose low-dose radioactive iodine (RAI) therapy. After the patient had been informed and given his consent, he received an initial dose of 20 mCi (740 MBq) of iodine-131 administered in liquid form. Scintigraphy with single photon emission computed tomography (SPECT)/CT acquisitions was performed the same day, showing uptake of ectopic thyroid tissue in the trachea (Figure 2).

**Figure 3:**
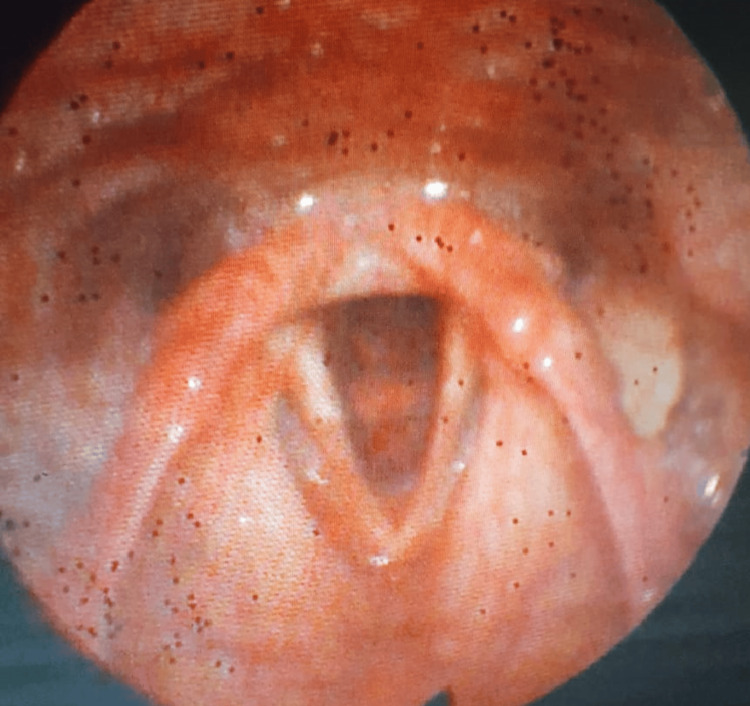
Post-treatment fibroscopic view showing the clearance of the upper airway and the complete resolution of the pathological process.

An ulterior control with scintigraphic technetium scan (99mTc) was performed three months later and showed a reduction in fixation intensity, consistent with the good evolution of the morphological aspect of the ectopic tissue as assessed by nasofibroscopy. A second dose was administered six months after the first dose. The final control with nasofibroscopy showed the complete resolution of the intra-tracheal mass (Figure 3).

The patient’s follow-up examination was favorable. The tracheotomy was re-established and the patient resumed his professional life as a teacher as normal. He developed hypothyroidism and was put on hormone replacement therapy.

## Discussion

The thyroid gland appears starting the third week of intrauterine life between the first and second branchial arches, in the form of epithelial proliferation of the endodermal lining on the floor of the pharyngeal intestine "the foramen cecum." The thyroid body then continues to migrate caudally and ventrally in relation to the hyoid bone and the cartilages of the larynx, reaching its final position in front of the trachea in the seventh week [2]. Ectopic thyroid tissue is the result of a lack of thyroid migration, not only along the thyroglossal duct but also in subdiaphragmatic organs such as the gallbladder and adrenal glands. The exact mechanism of thyroid morphogenesis is unknown [3,4].

In about one in 200,000 healthy people and one in 6,000 people with thyroid disease, the thyroid gland fails to descend correctly through the foramen cecum and it is four times more common in women [5,6]. The average age at which ectopy is discovered is 40.5 years, with extremes ranging from three months to 50 years [7].

The most common location of ectopic thyroid tissue is the base of the tongue, specifically the foramen cecum area, accounting for approximately 90% of reported cases. In 70-75% of cases, the lingual thyroid gland is the only thyroid tissue present. The most common symptoms associated with lingual thyroid growth include difficulty swallowing, dysphonia, foreign body sensation, coughing, snoring, sleep apnea, and in the most severe cases, airway obstruction and bleeding. Patients may also be asymptomatic. Therefore, lingual thyroid may have been discovered incidentally after investigation of nonthyroid-related symptoms. Regarding thyroid function, most patients with lingual thyroid have hypothyroidism and often do not have a thyroid gland in situ. They may also be euthyroid, even in the absence of an orthotopic thyroid [8].

However, the location of thyroid tissue has been described more rarely in the intratracheal, submaxillary, prehyoid, peripharyngeal, and mediastinal regions, and more exceptionally in the heart, esophagus, diaphragm, and ovaries [9]. Double thyroid ectopias are a very rare anomaly, accounting for around 9% of all thyroid ectopias [10]. In most of these cases, the orthotopic thyroid gland is usually in place and patients are euthyroid [8].

As was the case in our patient, the intratracheal thyroid is a very rare location for the ectopic thyroid gland described in the literature. Plausible explanations for this entity could be that the trachea and its ring of cartilage separate the developing thyroid gland, or that thyroid tissue grows into the tracheal lumen. The latter is due to a developmental defect in the interstitial tissue between the thyroid gland and the trachea, causing the original thyroid gland to adhere to the trachea [11]. Endotracheal ectopic thyroid disease can occur at any age but mainly occurs between the ages of 30 and 50 years, especially in women [12]. Patients typically present with cough, dysphagia, dyspnea, hemoptysis, and stridor, resulting from life-threatening upper airway obstruction, or may be asymptomatic. A normally functioning orthotopic thyroid usually coexists and, as a result, patients are euthyroid [11,12].

There is no consensus on the optimal treatment strategy for thyroid ectopia, possibly due to the rarity of this clinical condition. Most authors agree that the surgical treatment of ectopic thyroid glands in the neck (mainly lingual, sublingual, submandibular, and lateral cervical) depends on its size and local symptoms (respiratory obstruction, dysphagia, and dysphonia), among other parameters, such as the patient's age, functional status of the thyroid gland, and complications of the mass (ulceration, bleeding, cystic degeneration, or malignancy) [8]. Considering the possibility of malignant transformation, some recommend complete surgical resection [13]. In completely asymptomatic and euthyroid cases, regular follow-up is recommended to detect mass growth or the appearance of complications [8]. For mild symptoms and hypothyroidism, levothyroxine replacement therapy may be effective and result in significant mass reduction.

However, RAI for ectopic thyroid appears to be a safe and effective strategy that can completely resolve symptoms and could be an alternative to surgery when the latter is contraindicated or refused by the patient [1]. In our patient's case, surgical excision was refused by several surgeons. The multidisciplinary consultation meeting between the otorhinolaryngologist, thoracic surgeon, and nuclear physician proposed low-dose radioiodine therapy.

Low-dose RAI is the treatment of benign thyroid disorders with iodine-131. It was introduced in 1941 for the treatment of hyperthyroidism. Iodine-131 is an effective treatment that is easy to use, inexpensive, and ambulatory. The effectiveness of internal radiotherapy with RAI results from the high level of absorbed dose (D) in Gray (Gy) that it is possible to deliver to the thyroid cells and the relative tissue specificity of the irradiation [14,15].

## Conclusions

Intra-tracheal thyroid ectopy is a rare anomaly caused by defective migration of the thyroid gland during embryonic development. It requires a reduction in size when progressive hypertrophy of the ectopic thyroid tissue causes life-threatening obstructive or compressive oropharyngeal signs. Surgery has traditionally been used for the therapeutic management of this clinical entity. In certain cases where surgery is not feasible, treatment with iodine-131 is a safe and effective alternative for the removal of ectopic thyroid tissue, with marked improvement and resolution of tracheal obstruction.
